# Rational Design of Zika Virus Subunit Vaccine with Enhanced Efficacy

**DOI:** 10.1128/JVI.02187-18

**Published:** 2019-08-13

**Authors:** Wanbo Tai, Jiawei Chen, Guangyu Zhao, Qibin Geng, Lei He, Yuehong Chen, Yusen Zhou, Fang Li, Lanying Du

**Affiliations:** a New York Blood Center, Lindsley F. Kimball Research Institute, New York, New York, USA; b State Key Laboratory of Pathogen and Biosecurity, Beijing Institute of Microbiology and Epidemiology, Beijing, China; c Department of Veterinary and Biomedical Sciences, College of Veterinary Medicine, University of Minnesota, Saint Paul, Minnesota, USA; d Institute of Medical and Pharmaceutical Sciences, Zhengzhou University, Zhengzhou, China; University of North Carolina at Chapel Hill

**Keywords:** Zika virus, domain III, envelope protein, epitope shielding, glycan probe, intrinsic limitation of subunit vaccine designs, vaccine efficacy

## Abstract

Zika virus (ZIKV) infection in pregnant women can lead to fetal deaths and malformations. We have previously reported that ZIKV envelope protein domain III (EDIII) is a subunit vaccine candidate with cross-neutralization activity; however, like many other subunit vaccines, its efficacy is limited. To improve the efficacy of this subunit vaccine, we identified a nonneutralizing epitope on ZIKV EDIII surrounding residue 375, which is buried in the full-length envelope protein but becomes exposed in recombinant EDIII. We then shielded this epitope with an engineered glycan probe. Compared to the wild-type EDIII, the mutant EDIII induced significantly stronger neutralizing antibodies in three mouse strains and also demonstrated significantly improved efficacy by fully protecting mice, particularly pregnant mice and their fetuses, against high-dose lethal ZIKV challenge. Moreover, the mutant EDIII immune sera significantly enhanced the passive protective efficacy by fully protecting mice against lethal ZIKV challenge; this passive protection was positively associated with neutralizing antibody titers. We further showed that the enhanced efficacy of the mutant EDIII was due to the shielding of the immunodominant nonneutralizing epitope surrounding residue 375, which led to immune refocusing on the neutralizing epitopes. Taken together, the results of this study reveal that an intrinsic limitation of subunit vaccines is their artificially exposed immunodominant nonneutralizing epitopes, which can be overcome through glycan shielding. Additionally, the mutant ZIKV protein generated in this study is a promising subunit vaccine candidate with high efficacy in preventing ZIKV infections in mice.

**IMPORTANCE** Viral subunit vaccines generally show low efficacy. In this study, we revealed an intrinsic limitation of subunit vaccine designs: artificially exposed surfaces of subunit vaccines contain epitopes unfavorable for vaccine efficacy. More specifically, we identified an epitope on Zika virus (ZIKV) envelope protein domain III (EDIII) that is buried in the full-length envelope protein but becomes exposed in recombinant EDIII. We further shielded this epitope with a glycan, and the resulting mutant EDIII vaccine demonstrated significantly enhanced efficacy over the wild-type EDIII vaccine in protecting animal models from ZIKV infections. Therefore, the intrinsic limitation of subunit vaccines can be overcome through shielding these artificially exposed unfavorable epitopes. The engineered EDIII vaccine generated in this study is a promising vaccine candidate that can be further developed to battle ZIKV infections.

## Abstract (precis)

Viral subunit vaccines generally show low efficacy. In this study, we revealed an intrinsic limitation of subunit vaccine designs: artificially exposed surfaces of subunit vaccines contain epitopes unfavorable for vaccine efficacy. More specifically, we identified an epitope on Zika virus (ZIKV) envelope protein domain III (EDIII) that is buried in the full-length envelope protein but becomes exposed in recombinant EDIII. We further shielded this epitope with a glycan, and the resulting mutant EDIII vaccine demonstrated significantly enhanced efficacy over the wild-type EDIII vaccine in protecting animal models from ZIKV infections. Therefore, the intrinsic limitation of subunit vaccines can be overcome through shielding these artificially exposed unfavorable epitopes. The engineered EDIII vaccine generated in this study is a promising vaccine candidate that can be further developed to battle ZIKV infections.

## INTRODUCTION

Zika virus (ZIKV) is a member of the *Flavivirus* genus in the *Flaviviridae* family. Other members of the genus include dengue virus (DENV), West Nile virus (WNV), yellow fever virus (YFV), and Japanese encephalitis virus (JEV) (1, 2). ZIKV causes neurological diseases such as Guillain-Barré syndrome and congenital Zika syndrome (symptoms include microcephaly, brain abnormalities, and other congenital malformations) (3–6). Although several ZIKV vaccines are currently in clinical trials (7–9), no vaccine has been approved by the FDA to be used in humans.

The genome of ZIKV is a single-stranded positive-sense RNA and encodes a number of structural and nonstructural proteins (10, 11). The envelope (E) protein is a major structural protein and guides viral entry into host cells by first binding to a host receptor and then fusing viral and host membranes. It is anchored on viral envelopes and forms a dimer. Each monomer contains multiple structural domains: domain I (EDI), domain II (EDII), domain III (EDIII), the stem region, and the transmembrane domain (TM) (12, 13). The E protein is a main inducer of the host immune responses. Both the full-length E protein and its individual domains are prime targets for subunit vaccine design (14–19). Among these domains, EDI and EDII, but not EDIII, can trigger antibody-enhanced ZIKV entry (20–22). EDIII plays a critical role in viral entry by binding to host receptor(s). Because of its safety, EDIII has been our focus for subunit vaccine development. We previously showed that a ZIKV EDIII-based recombinant subunit vaccine is effective in eliciting long-term and broad-spectrum neutralizing antibodies against divergent ZIKV strains (23). However, its efficacy is not optimal. How to improve the efficacy of this subunit vaccine is key to its potential contribution to prevention of ZIKV infections.

Recombinant subunit vaccines have better safety over attenuated viral vaccines because unlike attenuated vaccines, subunit vaccines do not contain any residual infectivity. However, subunit vaccines often show insufficient efficacy. Structure-based vaccine designs have been intensively pursued to improve the efficacy of subunit vaccines, aiding battles against various viral diseases (24–26). In these vaccine designs, the envelope proteins of HIV, influenza virus, and non-ZIKV flaviviruses are modified to preserve specific neutralizing epitopes while subtracting or masking epitopes that potentially induce antibody-dependent enhancement effects or other unfavorable immune responses (24, 25, 27, 28). Trying to understand the generally low efficacy of viral subunit vaccines, we recently identified an intrinsic limitation of recombinant subunit vaccines, using Middle East respiratory syndrome (MERS) coronavirus (MERS-CoV) as a model system. That is, subunit vaccines are part of a large viral envelope protein or a virus particle, and hence much of their surface areas are buried; however, when a recombinant subunit vaccine is made, the previously buried surface areas become artificially exposed, and they contain immunodominant nonneutralizing epitopes that distract the host immune system from reacting to neutralizing epitopes (29). We also developed a neutralizing immunogenicity index (NII) to quantitatively characterize the contribution of each of these epitopes to the overall immunogenicity of the subunit vaccine (29). In spite of their initial success in MERS-CoV subunit vaccine designs, the concept of the intrinsic limitation of viral subunit vaccines and the strategy of using NII to quantitatively characterize epitopes have not been extended to subunit vaccine designs targeting another virus.

In this study, we analyzed the tertiary structure of ZIKV EDIII, identified an artificially exposed epitope on the vaccine surface, shielded it with a glycan probe, characterized its NII, and measured the efficacy of the mutant EDIII in protecting mice and their fetuses from ZIKV infections. We further investigated the molecular mechanism for the enhanced efficacy of the mutant EDIII. Overall, this study has firmly established and characterized an intrinsic limitation of viral subunit vaccines and pointed out a way to overcome this limitation. It has important implications for the design and development of novel viral subunit vaccines. The mutant ZIKV EDIII generated in this study can be a promising subunit vaccine candidate for preventing ZIKV infections in humans.

## RESULTS

### Identification and masking of a nonneutralizing epitope on ZIKV EDIII.

**FIG 1:**
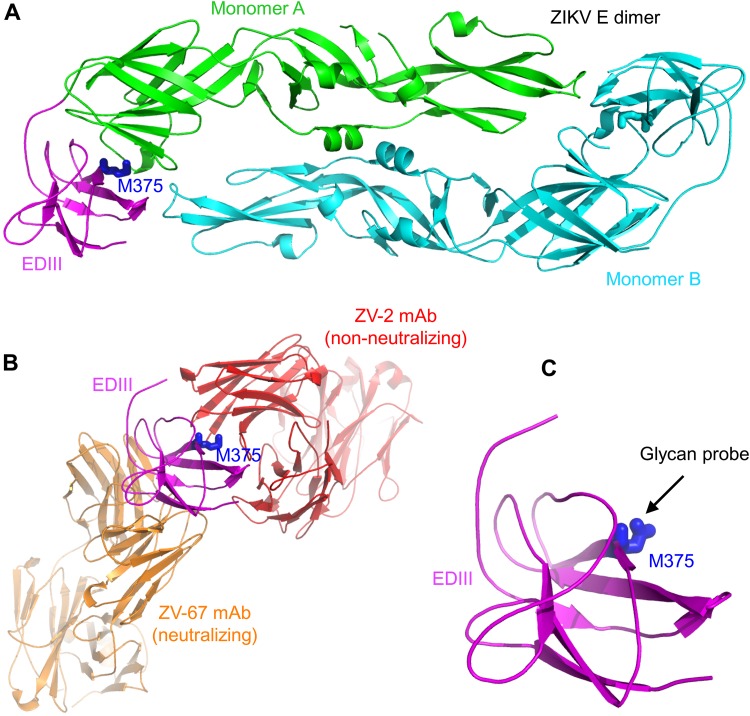
Structure-based design of ZIKV EDIII vaccine with enhanced efficacy. (A) Crystal structure of ZIKV E protein dimer (PDB ID 5LBV) (30). The two monomeric subunits are colored green and cyan. EDIII of monomeric subunit A is colored in magenta. Residue 375 of EDIII is shown in sticks and colored blue. (B) Crystal structures of ZIKV EDIII complexed with MAbs. Neutralizing MAb ZV-67 is colored in orange (PDB ID 5KVG) (10). Nonneutralizing MAb ZV-2 is colored in red (PDB ID 5KVD) (10). (C) Introducing an N-linked glycosylation site to residue 375 of EDIII (after introducing double mutations M375N/E377T to EDIII).

**FIG 2:**
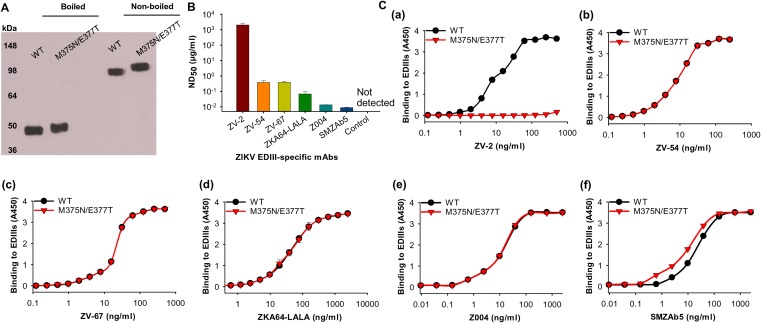
Characterization of mutant ZIKV EDIII vaccine with residue 375 shielded by a glycan probe. **(**A**)** ZIKV mutant (M375N/E377T) EDIII protein detected using Western blotting. ZIKV wild-type (WT) EDIII was included as a control. Proteins (5 μg) were analyzed using ZIKV E-His-specific mouse polyclonal antibodies (1:1,000) and HRP-conjugated goat anti-mouse IgG (1:5,000). Boiled and nonboiled protein samples are shown. Molecular weight markers are indicated on the left. (B) Neutralizing activity of ZIKV EDIII-specific MAbs against ZIKV. Mouse mAbs ZV-2 and ZV-54, as well as human MAbs ZV-67, ZKA64-LALA, Z004, and SMZAb5, were detected for neutralizing activity against ZIKV strain R103451 using the plaque reduction neutralization test. PBS was included as a control. Neutralizing activity is expressed as mean 50% neutralization dose (ND_50_) ± SEM of quadruplicate wells. (C) Antigenicity of mutant ZIKV EDIII vaccine. ELISA was performed to test the binding of mutant M375N/E377T protein to ZIKV EDIII-specific MAbs ZV-2 (a), ZV-54 (b), ZV-67 (c), ZKA64-LALA (d), Z004 (e), and SMZAb5 (f). ZIKV EDIII wild-type (WT) protein was used as a comparison. Error bars indicate SEM (*n* = 4). The experiments were repeated three times, and similar results were obtained.

To identify potentially immunodominant nonneutralizing epitopes on ZIKV EDIII, we analyzed the crystal structure of ZIKV E protein dimer (PDB identifier [ID] 5LBV) (30). We found a patch of surface area on EDIII that is buried in the full-length E protein dimer but becomes exposed in recombinant EDIII. Met375 is located in the center of this patch and protrudes from a bent β-strand (Fig. 1A and B). Hence, we engineered a glycan probe onto the epitope surrounding residue 375 (i.e., epitope 375) (Fig. 1C). To this end, we introduced double mutations M375N/E377T to EDIII, which changed residue 375 to an N-linked glycosylation site. We expressed and purified the mutant EDIII, along with wild-type EDIII, in mammalian 293T cells. Both proteins contain a C-terminal Fc tag and were purified to homogeneity. The results from Western blotting showed that both proteins ran as dimers without boiling (due to the Fc dimeric tag and incomplete denaturing) and as monomers after boiling (due to complete denaturing). The mutant EDIII ran slower and with a slightly larger molecular weight than wild-type EDIII (Fig. 2A), indicating that the mutant EDIII was glycosylated after addition of a glycan probe at residue 375.

To characterize the conformation of the mutant ZIKV EDIII, we investigated the binding interactions between ZIKV EDIII (wild type or mutant) and EDIII-specific monoclonal antibodies (MAbs) using enzyme-linked immunosorbent assay (ELISA). Among the MAbs used in this study, it has been shown previously that ZV-54, ZV-67, ZKA64-LALA, Z004, and SMZAb5 can potently neutralize ZIKV infections in mice and/or *in vitro*, whereas ZV-2 has no neutralizing activity *in vitro* (*in vitro* activities were characterized using a plaque-based neutralization assay) (Fig. 2B) (10, 23, 31, 32). Our results showed that the mutant EDIII bound to ZV-54, ZV-67, ZKA64-LALA, Z004, and SMZAb5 but did not bind to ZV-2. In comparison, wild-type EDIII bound to all of the six MAbs (Fig. 2C). The crystal structures of ZIKV E protein complexed with several of the MAbs have been determined previously by other groups (10, 31). Based on these previously determined structures, we analyzed the binding sites of these MAbs on EDIII. The crystal structures of ZIKV E protein complexed with ZV-67 and of ZIKV EDIII complexed with ZV-2 revealed that ZV-67 binds to an area that is exposed on the E protein dimer and a likely receptor-binding site, whereas ZV-2 binds to an area centering epitope 375 that is buried on the E protein dimer but becomes artificially exposed on the recombinant EDIII (Fig. 1B) (10). Structural analysis of EDIII complexed with Z004 identified the lateral ridge (away from epitope 375) of EDIII as its recognizing epitope (31). The structural bases for the binding of ZV-54, ZKA64-LALA, and SMZAb5 are not known, but these MAbs may also bind to functionally important regions on EDIII and away from epitope 375. Overall, these data further confirm that the mutant EDIII has successfully incorporated a glycan at residue 375, which blocked the binding of a nonneutralizing MAb ZV-2; they also reveal that despite the introduced glycan, the mutant EDIII retains its native structural conformation and antigenicity by binding to five different neutralizing MAbs.

### Measurement of neutralizing immunogenicity index of EDIII epitope 375.

**FIG 3:**
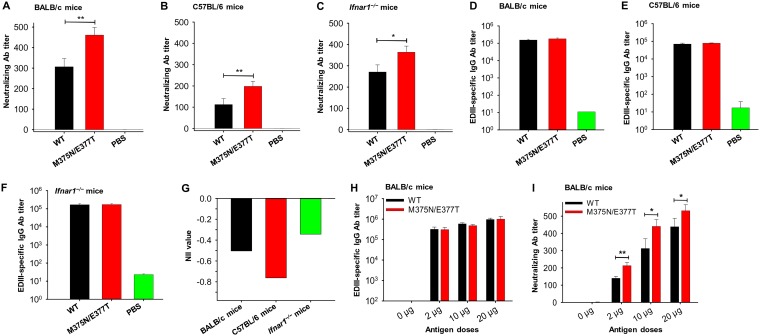
Characterization of IgG antibodies and neutralizing antibodies induced by mutant ZIKV EDIII vaccine. BALB/c, C57BL/6, and *Ifnar1^−/−^* mice were immunized with the wild-type (WT) and mutant (M375N/E377T) ZIKV EDIII vaccines. Sera were collected on day 10 post-2nd immunization for detection of neutralizing antibodies against ZIKV strain R103451 using the plaque reduction neutralization test (A to C) or IgG antibodies (D to F). PBS was included as a control. Neutralizing activity is expressed as 50% plaque reduction neutralizing antibody titer (PRNT_50_). The data are presented as mean PRNT_50_ ± SEM of quadruplicate wells of 2-fold serially diluted sera (pooled from 6 mice in each group). Significant differences between the WT and mutant EDIII proteins are shown (*, *P* < 0.05; **, *P* < 0.01). The IgG antibody titers are expressed as the endpoint dilutions that remain positively detectable. (G) Measured neutralizing immunogenicity index (NII) for mutant EDIII protein using serum neutralizing antibody titers (PRNT_50_) from BALB/c, C57BL/6, and *Ifnar1^−/−^* mice described above. The NII value was calculated based on the formula (PRNT_50-WT_ – PRNT_50-mutant_)/PRNT_50-WT_, where PRNT_50-WT_ and PRNT_50-mutant_ represent PRNT_50_ neutralizing antibody titers for the wild-type and mutant EDIII proteins, respectively. In a separate experiment, BALB/c mice were further immunized with different doses of above WT and mutant ZIKV EDIII proteins. Sera were collected on day 10 post-2nd immunization for detection of IgG (H) and neutralizing antibodies against ZIKV strain R103451 (I) as described above. The IgG antibody titers are expressed as the endpoint dilutions that remain positively detectable. Neutralizing antibody titers are expressed as PRNT_50_. The data are presented as means ± SEM of quadruplicate wells of 2-fold serially diluted sera (pooled from 5 mice in each group). The experiments were repeated three times, and similar results were obtained.

We previously defined the neutralizing immunogenicity index (NII) of an epitope as the contribution of the epitope to the overall neutralizing immunogenicity of the vaccine (29). For epitope 375 on ZIKV EDIII, the NII can be calculated as the difference between the neutralizing immunogenicity of wild-type EDIII and that of the mutant EDIII, divided by the neutralizing immunogenicity of wild-type EDIII. To measure the NII of epitope 375 on ZIKV EDIII, we immunized mice with wild-type EDIII and mutant EDIII individually and measured the neutralizing antibody titers of the induced sera. The immunized mice included immunocompetent BALB/c and C57BL/6 mice as well as immunocompromised interferon-α/β receptor (IFNAR)-deficient (*Ifnar1^−/−^*) mice (C57BL/6 background). The results showed that compared to the wild-type EDIII, the mutant EDIII induced significantly higher titers of anti-ZIKV neutralizing antibodies (Fig. 3A to C) but similar titers of ZIKV EDIII-specific IgG antibodies in all of the three types of immunized mice (Fig. 3D to F). These data suggest that epitope 375 makes a negative contribution to the overall neutralizing immunogenicity of EDIII; furthermore, masking epitope 375 does not affect the total IgG antibody production but instead increases the overall neutralizing immunogenicity of the vaccine. We also calculated the NII of epitope 375. The values of NII varied slightly in different mice: −0.50 in BALB/c, −0.76 in C57BL/6, and −0.34 in *Ifnar1^−/−^* mice (Fig. 3G). The negative NII confirms the negative contribution of epitope 375 to the overall neutralizing immunogenicity of the vaccine. The values for NII suggest that masking epitope 375 increases the overall neutralizing immunogenicity of the vaccine by 50%, 76%, and 34% [calculated using formula (PRNT_50-WT_ – PRNT_50-mutant_)/PRNT_50-WT_ × 100, where PRNT_50-WT_ is 50% plaque reduction neutralization titer for the wild type], respectively, in BALB/c, C57BL/6, and *Ifnar1^−/−^* mice. Therefore, these data reveal that epitope 375 is an immunodominant nonneutralizing epitope and that masking it can significantly improve the neutralizing immunogenicity of the EDIII vaccine.

To investigate how different antigen doses affect the neutralizing immunogenicity of the mutant ZIKV EDIII vaccine, BALB/c mice were divided into six groups and each group was immunized with one of three doses (2, 10, and 20 μg/mouse) of either the wild-type or mutant vaccine. The induced IgG and neutralizing antibodies were then compared. The results showed that for each of the doses tested, the wild-type and mutant EDIII vaccines induced the same amount of IgG antibody titers (Fig. 3H), while the mutant EDIII elicited significantly higher titers of anti-ZIKV neutralizing antibodies than wild-type EDIII did (Fig. 3I). In addition, vaccine doses were positively associated with the induced neutralizing antibody titers (Fig. 3I). These data suggest that immunization with increased doses of epitope 375-masked EDIII potentially improves the overall neutralizing immunogenicity of the vaccine.

**FIG 4:**
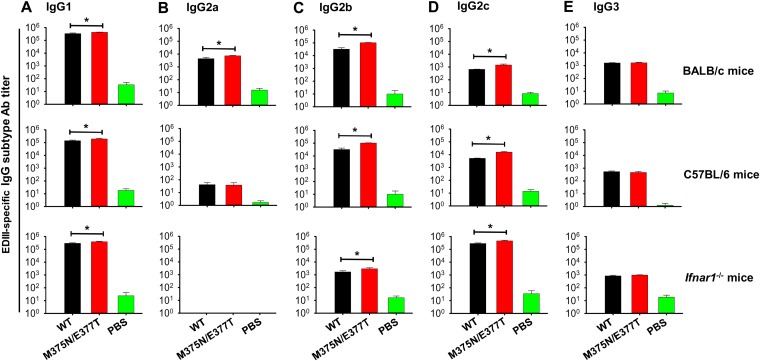
IgG subtype antibodies induced by the wild-type and mutant ZIKV EDIII vaccines. BALB/c, C57BL/6, and *Ifnar1^−/−^* mice were immunized with the wild-type and mutant (M375N/E377T) EDIII protein vaccines. Sera were collected on day 10 post-2nd immunization for detection of IgG subtype antibodies (A to E) that specifically targeted ZIKV EDIII protein. PBS was included as a control. The antibody titers are expressed in the form of the endpoint titers calculated as the reciprocal of the highest detectable dilution. The data are presented as mean ± SEM of mice in each group (*n* = 6). The experiments were repeated three times, and similar results were obtained. *, *P* < 0.05 between the wild-type and mutant EDIII vaccines.

To identify the predominant IgG subtypes and also to investigate whether epitope masking affects subtype production, sera of BALB/c, C57BL/6, and *Ifnar1^−/−^* mice immunized with the wild-type and mutant EDIII proteins were evaluated for their IgG subtype contents. The results showed that IgG1 was the predominant subtype induced by both vaccines in all mouse strains (Fig. 4A). On the other hand, no or low titers of IgG2a were detected in *Ifnar1^−/−^* and C57BL/6 mice (Fig. 4B), a low titer of IgG2b was detected in *Ifnar1^−/−^* mice (Fig. 4C), a low titer of IgG2c was detected in BALB/c mice (Fig. 4D), and a low titer of IgG3 was detected in all mouse strains tested (Fig. 4E). Overall, compared to the wild-type EDIII, the mutant EDIII induced more IgG1, IgG2a, IgG2b, and/or IgG2c subtypes in these mouse strains. These results demonstrate that although total IgG titers were similar in mice immunized by the wild-type and mutant EDIII vaccines, the associated IgG subtypes varied depending on the mouse strains tested.

### Enhanced efficacy of the mutant EDIII vaccine in protecting mice and their fetuses.

**FIG 5:**
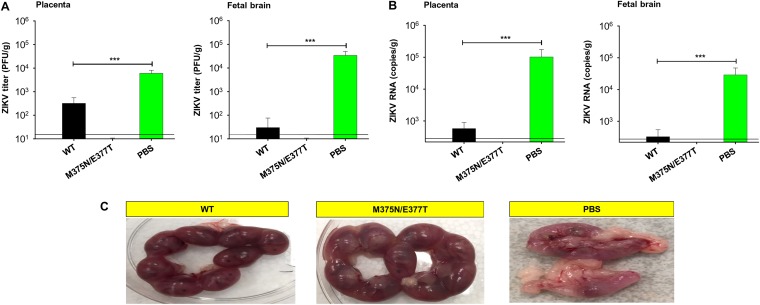
Characterization of the protective efficacy of mutant ZIKV EDIII vaccine in ZIKV-challenged pregnant BALB/c mice and their fetuses. Female BALB/c mice were immunized with the EDIII vaccine (wild type or mutant) or PBS control and mated with male BALB/c mice for pregnancy on day 10 post-2nd immunization. The pregnant mice (E10-E13) were challenged with ZIKV stain R103451 (2 × 10^5^ PFU) and detected for viral titers and viral RNAs in placenta and fetal brain using plaque (A) and quantitative reverse transcriptase PCR (qRT-PCR) (B) assays 6 days after challenge. Error bars indicate SEM (*n* = 6). ***, *P* < 0.001. The detection limit for plaque assay was 20 PFU/g, and for qRT-PCR was 2.5 × 10^2^ RNA copies/g, of tissue. (C) Morphology of uteri and embryos in immunized pregnant BALB/c mice 6 days after ZIKV challenge.

We investigated the efficacy of the mutant ZIKV EDIII vaccine in protecting immunocompetent pregnant mice and their fetuses from ZIKV infection (immunocompetent adult mice, such as BALB/c, are nonlethal models for ZIKV infection). To this end, we immunized BALB/c mice with either the wild-type or mutant EDIII vaccine and challenged the pregnant mice with ZIKV. We then collected placenta and fetal brain 6 days postchallenge and measured the titers and RNA copies of ZIKV in these samples, respectively, using plaque and quantitative reverse transcription-PCR (qRT-PCR) assays. Embryos were also collected for comparison of fetal status. The results showed that in contrast to the wild-type-EDIII-treated group, the mutant-EDIII-immunized pregnant mice had undetectable viral titers and RNA copies in their placentas and fetal brain (Fig. 5A and B). Negative-control pregnant mice, treated with phosphate-buffered saline (PBS), contained significantly higher viral titers and RNA copies in their tissues (Fig. 5A and B). In addition, there were no significant morphological changes in the uteri of the wild-type- and mutant-EDIII-immunized pregnant mice, and the embryos from these mice were in good condition. In contrast, the PBS-treated mice showed severe resorption in their uteri and the embryos from these mice were invisible (Fig. 5C). Collectively, compared to the wild-type EDIII vaccine, the mutant EDIII vaccine provides enhanced efficacy in protection of immunocompetent pregnant mice from ZIKV infection as evidenced by undetectable viral titers and RNA copies, although both the wild-type and mutant EDIII vaccines protect the fetuses of immunocompetent pregnant mice from ZIKV infection.

**FIG 6:**
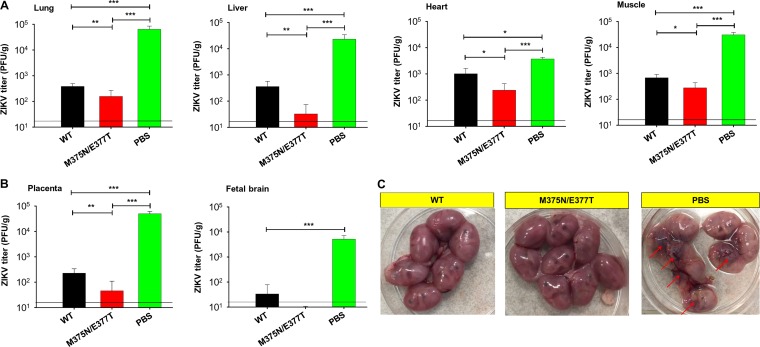
Characterization of the protective efficacy of mutant ZIKV EDIII vaccine in ZIKV-challenged pregnant *Ifnar1^−/−^* mice and their fetuses. *Ifnar1^−/−^* mice were immunized with the EDIII vaccine (wild-type or mutant) or PBS control and mated with male *Ifnar1^−/−^* mice for pregnancy on day 10 post-2nd immunization. The pregnant mice (E10-E13) were challenged with ZIKV strain R103451 (10^3^ PFU) and tested for viral titers using plaque assay in different tissues (including lung, liver, heart, and muscle [A] and placenta and fetal brain [B]) 6 days postchallenge. Error bars indicate SEM (*n* = 6). *, **, and ***, *P* < 0.05, 0.01, and 0.001, respectively. The detection limit for lung and heart was 25 PFU/g, and for liver, muscle, placenta, and fetal brain was 20 PFU/g, of tissue. (C) Morphology of uteri and embryos of immunized *Ifnar1^−/−^* pregnant mice 6 days after ZIKV challenge as described above. Arrows indicate fetal death.

**FIG 7:**
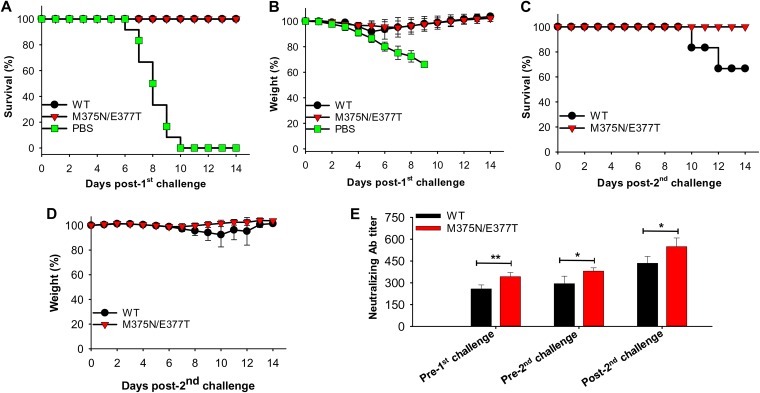
Enhanced protective efficacy of mutant ZIKV EDIII vaccine in ZIKV-challenged lethal *Ifnar1^−/−^* mouse model. Adult male *Ifnar1^−/−^* mice were immunized with the ZIKV WT and mutant (M375N/E377T) EDIII proteins, or PBS control, and challenged with ZIKV (R103451; 10^3^ PFU) 10 days post-2nd immunization to evaluate survival (A) and weight (B) for 14 days (*n* = 6). The surviving *Ifnar1^−/−^* mice in the WT and mutant M375N/E377T groups were further challenged with ZIKV (R103451; 5 × 10^4^ PFU) and observed for survival (C) and weight (D) as described above (*n* = 6). The percent weight in panels B and D represents the mean percent weight of all surviving mice at the indicated days after challenge. Error bars indicate SEM. (E) Neutralizing antibodies in the sera of mice before the 1st and 2nd challenges and post-2nd challenge against ZIKV strain R103451 by plaque reduction neutralization assay. The data are expressed as mean PRNT_50_ ± SEM of quadruplicate wells of 2-fold serially diluted sera pooled from 4 to 6 mice in each group. The experiments were repeated three times, and similar results were obtained.

We also evaluated the efficacy of the mutant ZIKV EDIII vaccine in protecting immunocompromised mice and their fetuses from ZIKV infection (immunocompromised mice are lethal models for ZIKV infection). To this end, we immunized female and male *Ifnar1^−/−^* mice with either the wild-type or mutant ZIKV EDIII vaccine. We then treated female and male mice differently. First, we challenged immunized female *Ifnar1^−/−^* pregnant mice with ZIKV, collected their tissues (including placenta and fetal brain) 6 days postchallenge, and measured the ZIKV titers in these tissues using the plaque assay. We also examined the embryos and uteri 6 days postchallenge. The results showed that compared to the wild-type-EDIII-treated group, the mutant-EDIII-immunized female *Ifnar1^−/−^* pregnant mice had significantly lower viral titers in their lung, liver, heart, muscle (Fig. 6A), and placenta and undetectable viral titers in fetal brain (Fig. 6B). There were no significant morphological changes in the uteri. Moreover, all embryos were in good condition in the wild-type- and mutant-EDIII-treated mice (Fig. 6C). In the negative-control group (i.e., PBS-treated female *Ifnar1^−/−^* pregnant mice), the viral titers were high in all the tested tissues and fetal brain, and there were significant morphological changes in the uteri, with severe fetal resorption or fetal death (Fig. 6). Second, we challenged adult male *Ifnar1^−/−^* mice with low-dose (10^3^ PFU) and high-dose (5 × 10^4^ PFU) ZIKV sequentially and observed them for survival and weight changes. The results showed that the male *Ifnar1^−/−^* mice, which were immunized with either the wild-type or mutant EDIII vaccine and then challenged with low-dose ZIKV, all survived 14 days post-1st challenge (Fig. 7A); their weight decreased slightly during days 4 to 7 postinfection but kept increasing steadily afterwards (Fig. 7B). In contrast, in the negative-control group (i.e., PBS-treated mice), all mice had at least 25% weight loss and were humanely euthanized 10 days postinfection (Fig. 7A and B). Moreover, the mutant-EDIII-immunized mice all survived the second, high-dose ZIKV challenge (100% survival) with no obvious weight loss. In contrast, only 67% of the wild-type-EDIII-immunized mice survived the second, high-dose ZIKV challenge, with considerable weight loss during days 7 to 12 postchallenge (Fig. 7C and D). Furthermore, neutralizing antibody titers in the mutant-EDIII-immunized mice were significantly higher than in the wild-type-EDIII-immunized mice before both the 1st and 2nd challenges, particularly after the 2nd challenge (Fig. 7E). Therefore, the enhanced protective efficacy of the mutant EDIII vaccine against the high-dose ZIKV challenge was likely due to the higher serum neutralizing antibody titers that it induced. Overall, the above data reveal that the mutant EDIII vaccine protects immunocompromised female pregnant mice and their fetuses from ZIKV infection more effectively than the wild-type EDIII vaccine (as evidenced by undetectable or significantly reduced viral titers), and it also protects immunocompromised adult male mice from high-dose ZIKV infection more effectively (as evidenced by improved survival rate and steadily maintained weight).

### Enhanced passive protection efficacy of the mutant-ZIKV-EDIII-induced mouse sera.

**FIG 8:**
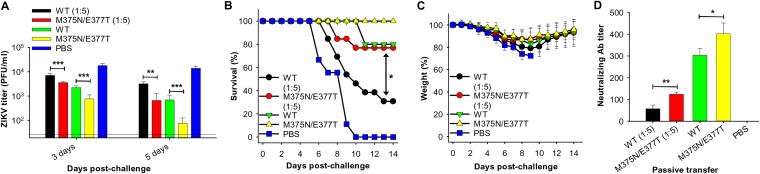
Characterization of the protective efficacy of the passively transferred sera from mutant-ZIKV-EDIII-vaccine-immunized mice. *Ifnar1^−/−^* mice were passively transferred with mutant (M375N/E377T) EDIII-immunized mouse sera. Six hours later, they were challenged with ZIKV strain R103451 (10^3^ PFU), followed by evaluation of serum viral titers on days 3 and 5 postchallenge, as well as survival and weight changes for 14 days. Mouse sera from WT-EDIII- or PBS-immunized mice were included as controls. (A) ZIKV titers in the sera of challenged mice collected on days 3 and 5 after ZIKV challenge. Error bars indicate SEM (*n* = 5). ** and ***, *P* < 0.01 and 0.001, respectively. The detection limit was 20 PFU/ml of sera. Survival (B) and weight (C) rates of serum transferred mice after ZIKV challenge were also recorded. Error bars indicate SEM (*n* = 5 to 13). * in panel B, *P* < 0.05 between WT and mutant EDIII protein groups (1:5 dilution). The percent weight in panel C represents the mean percent weight of all surviving mice on days indicated after challenge. (D) Characterization of neutralizing antibodies in the above sera against ZIKV challenge using plaque reduction neutralization test. Neutralizing activity is expressed as PRNT_50_. Error bars indicate SEM (*n* = 5). * and **, *P* < 0.05 and 0.01, respectively. The experiments were repeated three times, and similar results were obtained.

To understand the association between the enhanced protective efficacy of the mutant EDIII vaccine and the increased neutralizing antibody titers that it induced, passive protection experiments were performed using the wild-type or mutant-EDIII-induced mouse sera. To this end, sera were pooled from each group and normalized for equal EDIII-specific IgG titers. Adult *Ifnar1^−/−^* mice were passively transferred with the pooled sera (either undiluted or after 1:5 dilution). The mice were then challenged with ZIKV and their viremia, survival rate, and weight changes were subsequently monitored. The results showed that viremia was significantly lower in the mice passively transferred with the mutant-EDIII-induced sera than in those transferred with the wild-type-EDIII-induced sera (Fig. 8A). In addition, while 77% of the mice transferred with the diluted mutant-EDIII-induced sera survived the ZIKV challenge, mice transferred with undiluted sera all survived the ZIKV challenge (100% survival), both of which only showed slight weight loss (Fig. 8B and C). In comparison, for mice transferred with diluted or undiluted wild-type-induced sera, the survival rates were significantly lower or lower than for those with diluted or undiluted mutant-EDIII-induced sera, only about 31% and 80%, respectively; the former group also had significant weight loss during days 8 to 10 postchallenge (Fig. 8B and C). Moreover, the mutant-EDIII-induced sera had significantly higher anti-ZIKV neutralizing antibody titers than the wild-type-EDIII-induced sera (Fig. 8D). Negative-control mice transferred with the PBS-induced sera had a 0% survival rate at day 10 postchallenge; they also had the highest viral titers and most weight loss (Fig. 8). The above data indicate that compared to the wild-type-EDIII-induced sera, the mutant-EDIII-induced sera contained significantly higher neutralizing antibody titers as well as significantly enhanced passive protective efficacy against ZIKV challenge, which likely were positively associated with each other.

### Molecular mechanism for the enhanced efficacy of the mutant EDIII.

**FIG 9:**
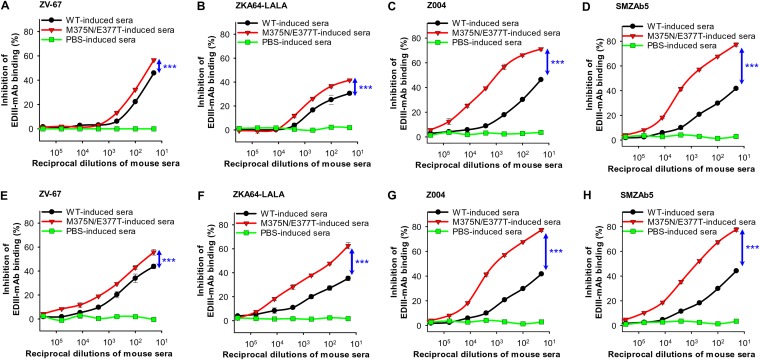
Masking of a nonneutralizing epitope surrounding residue 375 of ZIKV EDIII refocused neutralizing immunogenicity on neutralizing epitopes. ELISA was performed to detect the binding between human neutralizing MAbs and wild-type EDIII protein in the presence of mutant (M375N/E377T) EDIII-induced mouse sera. The MAbs include ZV-67 (A and E), ZKA64-LALA (B and F), Z004 (C and G), and SMZAb5 (D and H). The immune sera were from BALB/c (A to D) and *Ifnar1^−/−^* (E to H) mice. The WT-EDIII-induced mouse sera were used as a comparison. Mouse sera induced by PBS were used as a negative control. The percent inhibition in MAb-EDIII binding was calculated in the presence or absence of immune sera. Error bars indicate SEM (*n* = 4). Significant differences between WT- and mutant-EDIII-induced immune sera are shown. ***, *P* < 0.001. The experiments were repeated twice, and similar results were obtained.

To explore the molecular mechanism for the enhanced efficacy of the mutant EDIII vaccine, we examined competitive binding among EDIII, EDIII-specific MAbs, and EDIII-induced sera using ELISA. More specifically, we investigated the binding interactions between the wild-type EDIII and EDIII-specific neutralizing MAbs in the presence of the mutant-EDIII-induced mouse sera. The wild-type-EDIII-induced mouse sera were used as a comparison. The result showed that compared to the wild-type-EDIII-induced mouse sera, the mutant-EDIII-induced sera from both immunocompetent BALB/c mice (Fig. 9A to D) and immunocompromised *Ifnar1^−/−^* mice (Fig. 9E to H) blocked the binding between the wild-type EDIII and EDIII-specific neutralizing MAbs more effectively. As we showed earlier that the wild-type and mutant EDIII vaccines induced similar titers of total IgG antibodies (Fig. 3), the results from the competitive binding assay suggest that the mutant EDIII vaccine induced higher neutralizing antibody titers than the wild-type EDIII vaccine. Therefore, masking the immunodominant nonneutralizing epitope 375 (i.e., NII is negative and has a relatively large absolute value) triggered the host immune system to refocus on neutralizing, but less immunodominant, epitopes (i.e., NIIs are positive and have relatively small absolute values), accounting for the production of more neutralizing antibodies.

## DISCUSSION

ZIKV poses a significant threat to human health. Particularly, it has acquired the capability to cross the placenta and infect pregnant women and their fetuses, leading to severe consequences such as fetal neuropathology and fetal death (33, 34). Development of safe and effective vaccines to protect pregnant women and their fetuses is a high priority for the biomedical field. To date, a number of vaccine candidates have been tested in mouse and nonhuman primate models for ZIKV infection (16, 17, 35–37). Some of these vaccine candidates are currently in clinical trials (7–9, 38). In contrast to the traditional vaccines that typically focus on inactivated or attenuated virus particles, subunit vaccines contain only the antigenic components of the pathogen and hence are generally safer and more stable than traditional vaccines (http://www.who.int/vaccine_safety/initiative/tech_support/Part-2.pdf) (20, 39). The ZIKV E protein guides viral entry into cells and is a main inducer of the host immune response. It has been shown that unlike some domains of the E protein, recombinant ZIKV EDIII does not induce antibody-enhanced viral entry and hence is safe as a subunit vaccine in protecting mice from ZIKV challenge (23, 40). However, like many other recombinant subunit vaccines, ZIKV EDIII shows the following intrinsic limitation that affects its protective efficacy. As a domain of the ZIKV E protein, EDIII has large surface areas buried in the full-length dimeric E protein; when expressed individually as a recombinant EDIII, these previously buried surface areas become artificially exposed. The artificially exposed surface areas of EDIII may contain immunodominant nonneutralizing epitopes that distract the host immune system from reacting to neutralizing epitopes (e.g., epitopes in the receptor-binding regions). In this study, we aimed to overcome this intrinsic limitation of ZIKV EDIII. We first identified such an artificially exposed epitope on ZIKV EDIII around residue 375 and then introduced a glycan to cover this epitope (i.e., epitope 375). The goal was to shield epitope 375 from the host immune system, forcing the host immune system to refocus on neutralizing epitopes such as those in the receptor-binding regions.

We characterized the mutant ZIKV EDIII with epitope 375 shielded. Like wild-type EDIII, the mutant EDIII bound to EDIII-specific neutralizing MAbs through its receptor-binding regions, suggesting that the mutant EDIII retains its natural conformation. However, only the wild-type EDIII, and not the mutant EDIII, bound to an EDIII-specific nonneutralizing MAb targeting epitope 375, indicating that epitope 375 had been successfully glycosylated in the mutant EDIII. The increased molecular weight of the mutant EDIII also confirmed the successful glycosylation of epitope 375.

We measured the neutralizing immunogenicity index (NII) of epitope-375 in several mouse strains. The NIIs of epitope 375 in different mouse strains all were negative, suggesting that epitope 375 makes a negative contribution to the overall neutralizing immunogenicity of the EDIII vaccine. The NIIs of epitope 375 in different mouse strains vary from −0.34 to −0.76, suggesting that shielding of epitope 375 increases the overall neutralizing immunogenicity by 34% to 76%. Particularly, the absolute value of the NII for epitope 375 is lower in *Ifnar1^−/−^* mice (−0.34) than in BALB/c (−0.50) or C57BL/6 (−0.76) mice, suggesting that shielding of this epitope increases the overall neutralizing immunogenicity in immunocompromised *Ifnar1^−/−^* mice less than in immunocompetent BALB/c and C57BL/6 mice. Moreover, the neutralizing immunogenicity of the epitope 375-shielded mutant EDIII vaccine increases with the vaccine doses. We then examined the efficacy of the mutant EDIII in protecting mice from ZIKV challenges. Compared to the wild-type EDIII vaccine, the mutant EDIII vaccine protected female pregnant mice (both immunocompetent and immunocompromised mice) and their fetuses more effectively and also improved the survival rate of immunocompromised male mice against high-dose lethal ZIKV challenge. In addition, the mutant-EDIII-immunized mouse sera passively protected mice more effectively than the wild-type-EDIII-immunized mouse sera against lethal ZIKV challenge, and the protection was positively associated with serum neutralizing antibody titers. We further investigated the molecular mechanism behind the enhanced efficacy of the mutant EDIII vaccine. We found that shielding epitope 375 significantly increased the neutralizing antibody titers but maintained similar total IgG antibody titers in the immunized mice. Hence, shielding immunodominant nonneutralizing epitopes forces the host immune system to refocus on neutralizing epitopes of subunit vaccines. Studies on other flaviviruses, such as tick-borne encephalitis virus (TBEV), suggest that there may be variations in the specificity of antibody responses (including neutralizing antibody responses) between immunized mice and immunized (vaccinated or virus-infected) humans: while in mice antibody responses mainly target EDIII, in humans antibody responses mainly target EDI/EDII (41, 42). It is not clear whether this is the same case for ZIKV vaccines. Nevertheless, cautions should be taken for designing EDIII-based ZIKV subunit vaccines for human use.

It is worth noting that adjuvants play an important role in potentiating subunit vaccine-induced immune responses, including neutralizing antibodies. Aluminum hydroxide adjuvant may enhance overall immune responses and modulate specificity and functional activity of antibody responses that are induced by an EDIII-based TBEV vaccine in mice (41). Adjuvant System 04 (AS04), combining monophosphoryl lipid A (MPL) and aluminum salt, has been licensed for use in human vaccines (e.g., human papillomavirus and hepatitis B virus vaccines) with increased antibody titers (43, 44). Compared with aluminum adjuvant (hydroxide or salt) alone, the combination of MPL and aluminum salt may induce isotype-switched IgG antibody responses and significantly elevate total IgG and neutralizing antibodies as induced by subunit vaccines with an acceptable safety profile (45–47). Indeed, in this study, we found that the aluminum hydroxide and MPL adjuvant combination elicited high-titer neutralizing antibodies in conjugation with both the wild-type and mutant ZIKV EDIII vaccines. In addition, compared to the recombinant protein vaccines targeting EDIII of other flaviviruses with Freund’s adjuvants, the wild-type ZIKV EDIII vaccine with the aluminum hydroxide and MPL adjuvant combination seems to induce higher titers of neutralizing antibodies (48, 49). In general, as demonstrated in this and other studies, vaccines targeting ZIKV EDIII elicit high IgG antibody titers but relatively low neutralizing antibody titers (23, 40, 50), hence justifying the use of adjuvants. Consistent with previous data (51, 52), ZIKV EDIII vaccines elicited potent IgG1 subtype antibody responses in all mouse strains tested, suggesting that this antibody subtype might be associated with neutralizing antibody production. We also found that the aluminum hydroxide and MPL adjuvant combination helps the induction of IgG2a (for BALB/c mice), IgG2b, and IgG2c subtypes (for C57BL/6 and C57BL/6-based *Ifnar1^−/−^* mice), resulting in a more balanced immune response. Future studies will be needed to compare immunogenicity of ZIKV EDIII vaccines (wild type or mutant) in the presence of the aluminum hydroxide and MPL adjuvant combination, aluminum hydroxide alone, or MPL alone to determine whether the enhanced immunogenicity is associated with adjuvanticity of the combinational adjuvants. Such studies will also be helpful for comparing immunogenicity and efficacy of our vaccines with those of previous EDIII-based ZIKV or other flavivirus vaccines using same or different adjuvants.

To sum up, this study is the second successful application of the concept of NII in vaccine design, after the first application in MERS coronavirus subunit vaccine design (29). This study further establishes the following important principle in subunit vaccine designs. First, artificially exposed regions on viral subunit vaccines contain immunodominant nonneutralizing epitopes. Second, these epitopes limit the efficacy of viral subunit vaccines by distracting the host immune system from reacting to neutralizing epitopes. Third, shielding these epitopes can improve the efficacy of the vaccines by refocusing the host immune system on neutralizing epitopes. These principles can potentially be extended to many other viral subunit vaccines, such as vaccines against influenza and Ebola viruses. This study has also produced a safe and highly effective ZIKV subunit vaccine that holds promise to be developed into a successful vaccine to protect pregnant women and their fetuses. Therefore, the current study has important implications for both the battle against ZIKV and vaccine designs in general.

## MATERIALS AND METHODS

### Animals.

Four- to-6-week-old female BALB/c and C57BL/6 mice and 3- to 6-week-old male and female *Ifnar1^−/−^* mice were used in the study. The *Ifnar1^−/−^* mice (breeding pairs) on the C57BL/6 background were originally purchased from the Jackson Laboratory and were maintained for self-breeding in our animal facilities. The animal studies were carried out in strict accordance with the recommendations in the *Guide for the Care and Use of Laboratory Animals* (53). The protocols were approved by the Institutional Animal Care and Use Committee (IACUC) of the New York Blood Center (permit numbers 344.00 and 345.01).

### Expression and purification of recombinant proteins.

Recombinant ZIKV EDIII proteins were prepared as previously described (23, 54). Briefly, ZIKV EDIII protein containing residues 298 to 409 (i.e., wild type) was constructed based on ZIKV E (ZikaSPH2015 strain; GenBank accession number KU321639.1) fused with a C-terminal human IgG1 Fc tag. A mutant ZIKV EDIII protein containing a glycan probe surrounding residue 375 (i.e., M375N/E377T) was constructed by multisite mutagenesis kits (Thermo Fisher Scientific) using wild-type EDIII plasmid as the template. The recombinant proteins were transiently expressed in 293T cell culture supernatants and purified by protein A affinity chromatography (GE Healthcare).

### Animal immunization.

Purified ZIKV EDIII vaccines (wild type or mutant) were used to immunize mice as previously described (23, 55). Briefly, groups of six BALB/c, C57BL/6 (4- to 6-week-old), and *Ifnar1^−/−^* (3-week-old) mice were intramuscularly (i.m.) immunized with the EDIII vaccine (wild- type or mutant) (10 μg/mouse) or PBS control, plus a combination of aluminum hydroxide adjuvant (500 μg/mouse, vau-alu-250; InvivoGen) and monophosphoryl lipid A (MPL) adjuvant (10 μg/mouse, vac-mpla; InvivoGen). The immunized mice were boosted once with the same immunogens at 4 weeks, and sera were collected 10 days post-last immunization to test antibody responses and neutralizing antibodies. In addition, groups of five BALB/c mice (4 to 6 weeks old) were immunized and boosted with the EDIII vaccine (wild type or mutant) at 0, 2, 10, and 20 μg/mouse, respectively, in the presence of aluminum hydroxide and MPL adjuvant combination, followed by detection of IgG and neutralizing antibodies as described above.

### Western blotting.

The purified proteins were analyzed using Western blotting as previously described (23, 56). Briefly, proteins were first run on 10% Tris-glycine SDS-PAGE gels and were then transferred to nitrocellulose membranes for Western blot analysis. The transferred blot was blocked with 5% fat-free milk in PBS-Tween 20 (PBST) overnight at 4°C, followed by sequential incubation with ZIKV E-His protein-immunized mouse sera (1:1,000, laboratory stock) and horseradish peroxidase (HRP)-conjugated goat anti-mouse IgG (1:5,000) for 1 h at room temperature. The signal was visualized with ECL Western blot substrate buffer and Amersham Hyperfilm (GE Healthcare).

### ELISA.

The binding interactions between ZIKV EDIII proteins and EDIII-specific MAbs were characterized using ELISA as previously described (57). These MAbs include mouse MAbs ZV-2 (BEI Resources) and ZV-54, as well as human MAbs ZV-67, ZKA64-LALA, Z004, and SMZAb5 (Absolute Antibody) (10, 21, 31, 32). Briefly, ELISA plates were coated with the wild-type or mutant ZIKV EDIII protein overnight at 4°C and blocked with 2% fat-free milk in PBST for 2 h at 37°C. After three washes, the plates were sequentially incubated with serially diluted MAbs and HRP-conjugated anti-mouse IgG-Fab (for ZV-2 and ZV-54, 1:5,000; Thermo Fisher Scientific) or anti-human IgG-Fab (for ZV-67, ZKA64-LALA, Z004, and SMZAb5, 1:5,000; Abcam) antibody for 1 h at 37°C. The reaction was detected using the substrate 3,3′,5,5′-tetramethylbenzidine (Sigma) and stopped with 1 N H_2_SO_4_. Absorbance at 450 nm was measured using an ELISA plate reader (Tecan).

ZIKV EDIII-specific antibodies in mouse sera were tested using ELISA as described above except that the plates were coated with the wild-type ZIKV EDIII protein, followed by sequential incubation with serially diluted mouse sera and HRP-conjugated anti-mouse IgG (1:5,000), IgG1 (1:5,000), IgG2a (1:2,000), IgG2b (1:2,000), IgG2c (1:2,000), or IgG3 (1:2,000) (Thermo Fisher Scientific).

The competition between ZIKV EDIII-specific human MAbs (including ZV-67, ZKA64-LALA, Z004, or SMZAb5) and mutant EDIII-induced mouse sera for the binding of wild-type ZIKV EDIII was performed using ELISA as described above, except that the binding between wild-type EDIII and the MAbs (0.5 μg/ml) was tested in the presence of serially diluted mouse sera induced by the ZIKV EDIII vaccine (wild type or mutant) or PBS. The EDIII-MAb binding was measured by addition of HRP-conjugated anti-human IgG-Fab antibody (1:5,000) and subsequent enzymatic reaction.

### ZIKV plaque-forming assay and plaque reduction neutralization test.

ZIKV human strain R103451 (2015/Honduras) was amplified in Vero E6 cells for determination of viral titers using a standard plaque-forming assay. Viral titers in the tissues and/or sera from challenged mice were detected using the same approach. The ZIKV titers were detected from around 40 or 50 mg of tissue samples, or 50 μl of sera, so the detection limit was about 25 or 20 PFU/g of tissues, or 20 PFU/ml of sera. A plaque reduction neutralization test was carried out to measure neutralizing antibody titers in immunized mouse sera or activities of EDIII-specific MAbs as previously described (23). Briefly, ZIKV (100 PFU) was incubated with pooled mouse sera (at 2-fold serial dilutions from 1:10 to 1:2,560, quadruplicates of each dilution) or MAbs (4-fold serial dilutions from 1 μg/ml to 0.0005 μg/ml, quadruplicates of each dilution) for 1.5 h at 37°C, followed by addition of antibody-virus mixtures to Vero E6 cells and incubation for 1 h at 37°C. The cells were overlaid with medium (1% carboxymethyl cellulose in Dulbecco modified Eagle medium [DMEM] containing 2% fetal bovine serum [FBS]) and cultured at 37°C for 4 to 5 days before being stained with 0.5% crystal violet. PRNT_50_ and 50% neutralization dose (ND_50_) were calculated through dilutions and neutralizations of sera or MAbs at 50% plaque reduction using the CalcuSyn computer program (which is based on the median effect principle) (58–62). Specific PRNT_50_ and ND_50_ of sera or MAbs were obtained after inputting individual dilutions and neutralization values for each serum or MAb to the program.

### ZIKV challenge and protection evaluation.

ZIKV challenge experiments were carried out as previously described (23, 63). Briefly, 10 days post-last immunization, female *Ifnar1^−/−^* and BALB/c mice immunized with ZIKV EDIII (wild type or mutant) were mated with respective naive male mice within the same strains. Mice injected with PBS were included as controls. Pregnant female mice (E10 to E13) were intraperitoneally (i.p.) challenged with ZIKV strain R103451 (10^3^ PFU for *Ifnar1^−/−^* mice and 2 × 10^5^ PFU for BALB/c mice; 200 μl/mouse). ZIKV titers and RNA copies in tissues of adult mice as well as placenta and fetal brain (collected at 6 days postinfection) were detected using plaque-forming assay (described above) and qRT-PCR (described below), respectively. In a separate experiment, male *Ifnar1^−/−^* mice were (i.p.) challenged with ZIKV (R103451; 10^3^ PFU) 10 days post-last immunization and evaluated for survival and weight for another 14 days. Ten days after completion of the 1st challenge experiment, the surviving *Ifnar1^−/−^* mice in the wild-type or mutant ZIKV EDIII groups were further challenged with ZIKV (R103451; 5 × 10^4^ PFU) and observed for survival and weight as described above. Mice with ≥25% body weight loss were humanely euthanized.

### Passive protection.

Groups of 5 to 13 male and female *Ifnar1^−/−^* mice (5-6-week old) were injected (i.p.) with pooled sera (200 μl/mouse; normalized for equal ZIKV EDIII-specific IgG titers at 10^5^ or a 1:5 dilution in PBS) of wild-type- or mutant-EDIII-vaccine-immunized *Ifnar1^−/−^* mice collected before the 1st and 2nd ZIKV challenges. Six hours later, mice were i.p. challenged with ZIKV strain R103451 (10^3^ PFU). Mice injected with PBS-induced *Ifnar1^−/−^* mouse sera were included as controls. Neutralizing activity of passively transferred mouse sera was detected using the plaque reduction neutralization test. ZIKV titers were detected from sera collected at 3 days and 5 days postchallenge using the ZIKV plaque-forming assay. Mouse survival and weight were recorded for 14 days postchallenge. Mice with ≥25% body weight loss were humanely euthanized.

### qRT-PCR.

ZIKV RNA copies in sera and tissues of challenged mice were detected using qRT-PCR as previously described (23). Briefly, RNAs were extracted using QIAamp MinElute virus spin kit (for sera) (Qiagen) and RNeasy minikit (for tissues) (Qiagen) and quantified through one-step qRT-PCR using Power SYBR green PCR master mix, MultiScribe reverse transcriptase, and Ambion RNase inhibitor (Thermo Fisher Scientific) in a ViiA 7 Master Cycler PCR system (Thermo Fisher Scientific). The forward and reverse primers for the amplification were 5′-TTGGTCATGATACTGCTGATTGC-3′ and 5′-CCTTCCACAAAGTCCCTATTGC-3′.

A standard curve of qRT-PCR was established by 10-fold serial dilutions of a recombinant plasmid containing ZIKV membrane (M) and E genes. A linear standard curve at 10^1^ to 10^8^ RNA copies (correlation coefficient [*R*^2^] value > 0.98; detection limit = 10^1^ RNA copies) was selected for calculation of ZIKV RNA in the samples. The ZIKV RNA was purified from around 40 mg of tissue samples, so the detection limit was about 2.5 × 10^2^ RNA copies/g of tissues (64).

### Statistical analysis.

The values were presented as means with standard errors of the means (SEM). Statistical significance of antibody titers among different groups was calculated using the Mann-Whitney test. Statistical significance of viral titers among different groups, as well as serum inhibition, was calculated through Student’s two-tailed *t* test. Statistical significance of survival curves among different groups was calculated through the log rank test using GraphPad Prism statistical software.

## References

[1] Lazear HM, Diamond MS 2016 Zika virus: new clinical syndromes and its emergence in the Western Hemisphere. J Virol 90:4864–4875. doi:10.1128/JVI.00252-16. 26962217 PMC4859708

[2] Gatherer D, Kohl A 2016 Zika virus: a previously slow pandemic spreads rapidly through the Americas. J Gen Virol 97:269–273. doi:10.1099/jgv.0.000381. 26684466

[3] Kassavetis P, Joseph JB, Francois R, Perloff MD, Berkowitz AL 2016 Zika virus-associated Guillain-Barre syndrome variant in Haiti. Neurology 87:336–337. doi:10.1212/WNL.0000000000002759. 27164708

[4] Salinas JL, Walteros DM, Styczynski A, Garzon F, Quijada H, Bravo E, Chaparro P, Madero J, Acosta-Reyes J, Ledermann J, Arteta Z, Borland E, Burns P, Gonzalez M, Powers AM, Mercado M, Solano A, Sejvar JJ, Ospina ML 2017 Zika virus disease-associated Guillain-Barre syndrome—Barranquilla, Colombia 2015-2016. J Neurol Sci 381:272–277. doi:10.1016/j.jns.2017.09.001. 28991697

[5] Lucey D, Cummins H, Sholts S 2017 Congenital Zika syndrome in 2017. JAMA 317:1368–1369. doi:10.1001/jama.2017.1553. 28384812

[6] Driggers RW, Ho CY, Korhonen EM, Kuivanen S, Jaaskelainen AJ, Smura T, Rosenberg A, Hill DA, Debiasi RL, Vezina G, Timofeev J, Rodriguez FJ, Levanov L, Razak J, Iyengar P, Hennenfent A, Kennedy R, Lanciotti R, Du PA, Vapalahti O 2016 Zika virus infection with prolonged maternal viremia and fetal brain abnormalities. N Engl J Med 374:2142–2151. doi:10.1056/NEJMoa1601824. 27028667

[7] Gaudinski MR, Houser KV, Morabito KM, Hu Z, Yamshchikov G, Rothwell RS, Berkowitz N, Mendoza F, Saunders JG, Novik L, Hendel CS, Holman LA, Gordon IJ, Cox JH, Edupuganti S, Mcarthur MA, Rouphael NG, Lyke KE, Cummings GE, Sitar S, Bailer RT, Foreman BM, Burgomaster K, Pelc RS, Gordon DN, Demaso CR, Dowd KA, Laurencot C, Schwartz RM, Mascola JR, Graham BS, Pierson TC, Ledgerwood JE, Chen GL 2018 Safety, tolerability, and immunogenicity of two Zika virus DNA vaccine candidates in healthy adults: randomised, open-label, phase 1 clinical trials. Lancet 391:552–562. doi:10.1016/S0140-6736(17)33105-7. 29217376 PMC6379903

[8] Modjarrad K, Lin L, George SL, Stephenson KE, Eckels KH, De La Barrera RA, Jarman RG, Sondergaard E, Tennant J, Ansel JL, Mills K, Koren M, Robb ML, Barrett J, Thompson J, Kosel AE, Dawson P, Hale A, Tan CS, Walsh SR, Meyer KE, Brien J, Crowell TA, Blazevic A, Mosby K, Larocca RA, Abbink P, Boyd M, Bricault CA, Seaman MS, Basil A, Walsh M, Tonwe V, Hoft DF, Thomas SJ, Barouch DH, Michael NL 2018 Preliminary aggregate safety and immunogenicity results from three trials of a purified inactivated Zika virus vaccine candidate: phase 1, randomised, double-blind, placebo-controlled clinical trials. Lancet 391:563–571. doi:10.1016/S0140-6736(17)33106-9. 29217375 PMC5884730

[9] Tebas P, Roberts CC, Muthumani K, Reuschel EL, Kudchodkar SB, Zaidi FI, White S, Khan AS, Racine T, Choi H, Boyer J, Park YK, Trottier S, Remigio C, Krieger D, Spruill SE, Bagarazzi M, Kobinger GP, Weiner DB, Maslow JN 2017 Safety and immunogenicity of an anti-Zika virus DNA vaccine—preliminary report. N Engl J Med doi:10.1056/Nejmoa1708120. PMC6824915 34525286

[10] Zhao H, Fernandez E, Dowd KA, Speer SD, Platt DJ, Gorman MJ, Govero J, Nelson CA, Pierson TC, Diamond MS, Fremont DH 2016 Structural basis of Zika virus-specific antibody protection. Cell 166:1016–1027. doi:10.1016/j.cell.2016.07.020. 27475895 PMC4983199

[11] Dai L, Song J, Lu X, Deng YQ, Musyoki AM, Cheng H, Zhang Y, Yuan Y, Song H, Haywood J, Xiao H, Yan J, Shi Y, Qin CF, Qi J, Gao GF 2016 Structures of the Zika virus envelope protein and its complex with a flavivirus broadly protective antibody. Cell Host Microbe 19:696–704. doi:10.1016/j.chom.2016.04.013. 27158114

[12] Sirohi D, Chen Z, Sun L, Klose T, Pierson TC, Rossmann MG, Kuhn RJ 2016 The 3.8 Å resolution cryo-EM structure of Zika virus. Science 352:467–470. doi:10.1126/science.aaf5316. 27033547 PMC4845755

[13] Kostyuchenko VA, Lim EX, Zhang S, Fibriansah G, Ng TS, Ooi JS, Shi J, Lok SM 2016 Structure of the thermally stable Zika virus. Nature 533:425–428. doi:10.1038/nature17994. 27093288

[14] To A, Medina LO, Mfuh KO, Lieberman MM, Wong TAS, Namekar M, Nakano E, Lai CY, Kumar M, Nerurkar VR, Lehrer AT 2018 Recombinant Zika virus subunits are immunogenic and efficacious in mice. mSphere 3:e00576-17. doi:10.1128/mSphere.00576-17. 29359186 PMC5760751

[15] Garg H, Sedano M, Plata G, Punke EB, Joshi A 2017 Development of virus-like-particle vaccine and reporter assay for Zika virus. J Virol 91:e00834-17. doi:10.1128/JVI.00834-17. 28794019 PMC5625514

[16] Griffin BD, Muthumani K, Warner BM, Majer A, Hagan M, Audet J, Stein DR, Ranadheera C, Racine T, De La Vega MA, Piret J, Kucas S, Tran KN, Frost KL, De GC, Soule G, Scharikow L, Scott J, Mctavish G, Smid V, Park YK, Maslow JN, Sardesai NY, Kim JJ, Yao XJ, Bello A, Lindsay R, Boivin G, Booth SA, Kobasa D, Embury-Hyatt C, Safronetz D, Weiner DB, Kobinger GP 2017 DNA vaccination protects mice against Zika virus-induced damage to the testes. Nat Commun 8:15743. doi:10.1038/ncomms15743. 28589934 PMC5467228

[17] Dowd KA, Ko SY, Morabito KM, Yang ES, Pelc RS, Demaso CR, Castilho LR, Abbink P, Boyd M, Nityanandam R, Gordon DN, Gallagher JR, Chen X, Todd JP, Tsybovsky Y, Harris A, Huang YS, Higgs S, Vanlandingham DL, Andersen H, Lewis MG, De La Barrera R, Eckels KH, Jarman RG, Nason MC, Barouch DH, Roederer M, Kong WP, Mascola JR, Pierson TC, Graham BS 2016 Rapid development of a DNA vaccine for Zika virus. Science 354:237–240. doi:10.1126/science.aai9137. 27708058 PMC5304212

[18] Espinosa D, Mendy J, Manayani D, Vang L, Wang C, Richard T, Guenther B, Aruri J, Avanzini J, Garduno F, Farness P, Gurwith M, Smith J, Harris E, Alexander J 2018 Passive transfer of immune sera induced by a Zika virus-like particle vaccine protects AG129 mice against lethal Zika virus challenge. EBioMedicine 27:61–70. doi:10.1016/j.ebiom.2017.12.010. 29269041 PMC5828544

[19] Abbink P, Larocca RA, De La Barrera RA, Bricault CA, Moseley ET, Boyd M, Kirilova M, Li Z, Nganga D, Nanayakkara O, Nityanandam R, Mercado NB, Borducchi EN, Agarwal A, Brinkman AL, Cabral C, Chandrashekar A, Giglio PB, Jetton D, Jimenez J, Lee BC, Mojta S, Molloy K, Shetty M, Neubauer GH, Stephenson KE, Peron JPS, Zanotto PMDA, Misamore J, Finneyfrock B, Lewis MG, Alter G, Modjarrad K, Jarman RG, Eckels KH, Michael NL, Thomas SJ, Barouch DH 2016 Protective efficacy of multiple vaccine platforms against Zika virus challenge in rhesus monkeys. Science 353:1129–1132. doi:10.1126/science.aah6157. 27492477 PMC5237380

[20] Du L, Zhou Y, Jiang S 2017 The latest advancements in Zika virus vaccine development. Expert Rev Vaccines 16:951–954. doi:10.1080/14760584.2017.1363648. 28783378

[21] Stettler K, Beltramello M, Espinosa DA, Graham V, Cassotta A, Bianchi S, Vanzetta F, Minola A, Jaconi S, Mele F, Foglierini M, Pedotti M, Simonelli L, Dowall S, Atkinson B, Percivalle E, Simmons CP, Varani L, Blum J, Baldanti F, Cameroni E, Hewson R, Harris E, Lanzavecchia A, Sallusto F, Corti D 2016 Specificity, cross-reactivity, and function of antibodies elicited by Zika virus infection. Science 353:823–826. doi:10.1126/science.aaf8505. 27417494

[22] Jiang S, Du L 2019 Advances in the research and development of therapeutic antibodies against the Zika virus. Cell Mol Immunol 16:96–97. doi:10.1038/s41423-018-0043-x. 29802365 PMC6318303

[23] Tai W, He L, Wang Y, Sun S, Zhao G, Luo C, Li P, Zhao H, Fremont DH, Li F, Jiang S, Zhou Y, Du L 2018 Critical neutralizing fragment of Zika virus EDIII elicits cross-neutralization and protection against divergent Zika viruses. Emerg Microbes Infect 7:7. doi:10.1038/s41426-017-0007-8. 29362446 PMC5837162

[24] Kulp DW, Steichen JM, Pauthner M, Hu X, Schiffner T, Liguori A, Cottrell CA, Havenar-Daughton C, Ozorowski G, Georgeson E, Kalyuzhniy O, Willis JR, Kubitz M, Adachi Y, Reiss SM, Shin M, De VN, Ward AB, Crotty S, Burton DR, Schief WR 2017 Structure-based design of native-like HIV-1 envelope trimers to silence non-neutralizing epitopes and eliminate CD4 binding. Nat Commun 8:1655. doi:10.1038/s41467-017-01549-6. 29162799 PMC5698488

[25] Kulp DW, Schief WR 2013 Advances in structure-based vaccine design. Curr Opin Virol 3:322–331. doi:10.1016/j.coviro.2013.05.010. 23806515 PMC4102719

[26] Morens DM, Fauci AS 2013 Emerging infectious diseases: threats to human health and global stability. PLoS Pathog 9:E1003467. doi:10.1371/journal.ppat.1003467. 23853589 PMC3701702

[27] Pierson TC, Fremont DH, Kuhn RJ, Diamond MS 2008 Structural insights into the mechanisms of antibody-mediated neutralization of flavivirus infection: implications for vaccine development. Cell Host Microbe 4:229–238. doi:10.1016/j.chom.2008.08.004. 18779049 PMC2678546

[28] Pantophlet R, Burton DR 2003 Immunofocusing: antigen engineering to promote the induction of HIV-neutralizing antibodies. Trends Mol Med 9:468–473. doi:10.1016/j.molmed.2003.09.001. 14604823

[29] Du L, Tai W, Yang Y, Zhao G, Zhu Q, Sun S, Liu C, Tao X, Tseng CK, Perlman S, Jiang S, Zhou Y, Li F 2016 Introduction of neutralizing immunogenicity index to the rational design of MERS coronavirus subunit vaccines. Nat Commun 7:13473. doi:10.1038/ncomms13473. 27874853 PMC5121417

[30] Barba-Spaeth G, Dejnirattisai W, Rouvinski A, Vaney MC, Medits I, Sharma A, Simon-Loriere E, Sakuntabhai A, Cao-Lormeau VM, Haouz A, England P, Stiasny K, Mongkolsapaya J, Heinz FX, Screaton GR, Rey FA 2016 Structural basis of potent Zika-dengue virus antibody cross-neutralization. Nature 536:48–53. doi:10.1038/nature18938. 27338953

[31] Robbiani DF, Bozzacco L, Keeffe JR, Khouri R, Olsen PC, Gazumyan A, Schaefer-Babajew D, Avila-Rios S, Nogueira L, Patel R, Azzopardi SA, Uhl LFK, Saeed M, Sevilla-Reyes EE, Agudelo M, Yao KH, Golijanin J, Gristick HB, Lee YE, Hurley A, Caskey M, Pai J, Oliveira T, Wunder EA Jr, Sacramento G, Nery N Jr, Orge C, Costa F, Reis MG, Thomas NM, Eisenreich T, Weinberger DM, De Almeida ARP, West AP Jr, Rice CM, Bjorkman PJ, Reyes-Teran G, Ko AI, Macdonald MR, Nussenzweig MC 2017 Recurrent potent human neutralizing antibodies to Zika virus in Brazil and Mexico. Cell 169:597–609. doi:10.1016/j.cell.2017.04.024. 28475892 PMC5492969

[32] Magnani DM, Rogers TF, Beutler N, Ricciardi MJ, Bailey VK, Gonzalez-Nieto L, Briney B, Sok D, Le K, Strubel A, Gutman MJ, Pedreno-Lopez N, Grubaugh ND, Silveira CGT, Maxwell HS, Domingues A, Martins MA, Lee DE, Okwuazi EE, Jean S, Strobert EA, Chahroudi A, Silvestri G, Vanderford TH, Kallas EG, Desrosiers RC, Bonaldo MC, Whitehead SS, Burton DR, Watkins DI 2017 Neutralizing human monoclonal antibodies prevent Zika virus infection in macaques. Sci Transl Med 9:Eaan8184. doi:10.1126/scitranslmed.aan8184. 28978754 PMC6155977

[33] Strafela P, Vizjak A, Mraz J, Mlakar J, Pizem J, Tul N, Zupanc TA, Popovic M 2017 Zika virus-associated micrencephaly: a thorough description of neuropathologic findings in the fetal central nervous system. Arch Pathol Lab Med 141:73–81. doi:10.5858/arpa.2016-0341-SA. 27726416

[34] Castro MC, Han QC, Carvalho LR, Victora CG, Franca G 2018 Implications of Zika virus and congenital Zika syndrome for the number of live births in Brazil. Proc Natl Acad Sci U S A 115:6177–6182. doi:10.1073/pnas.1718476115. 29844186 PMC6004455

[35] Richner JM, Himansu S, Dowd KA, Butler SL, Salazar V, Fox JM, Julander JG, Tang WW, Shresta S, Pierson TC, Ciaramella G, Diamond MS 2017 Modified mRNA vaccines protect against Zika virus infection. Cell 168:1114–1125. doi:10.1016/j.cell.2017.02.017. 28222903 PMC5388441

[36] Guo Q, Chan JF, Poon VK, Wu S, Chan CC, Hou L, Yip CC, Ren C, Cai JP, Zhao M, Zhang AJ, Song X, Chan KH, Wang B, Kok KH, Wen Y, Yuen KY, Chen W 2018 Immunization with a novel human type 5 adenovirus-vectored vaccine expressing the premembrane and envelope proteins of Zika virus provides consistent and sterilizing protection in multiple immunocompetent and immunocompromised animal models. J Infect Dis 218:365–377. doi:10.1093/infdis/jiy187. 29617816

[37] Lopez-Camacho C, Abbink P, Larocca RA, Dejnirattisai W, Boyd M, Badamchi-Zadeh A, Wallace ZR, Doig J, Velazquez RS, Neto RDL, Coelho DF, Kim YC, Donald CL, Owsianka A, De LG, Kohl A, Gilbert SC, Dorrell L, Mongkolsapaya J, Patel AH, Screaton GR, Barouch DH, Hill AVS, Reyes-Sandoval A 2018 Rational Zika vaccine design via the modulation of antigen membrane anchors in chimpanzee adenoviral vectors. Nat Commun 9:2441. doi:10.1038/s41467-018-04859-5. 29934593 PMC6015009

[38] Cohen J 2018 Steep drop in Zika cases undermines vaccine trial. Science 361:1055–1056. doi:10.1126/science.361.6407.1055. 30213891

[39] Zhang N, Jiang S, Du L 2014 Current advancements and potential strategies in the development of MERS-Cov vaccines. Expert Rev Vaccines 13:761–774. doi:10.1586/14760584.2014.912134. 24766432 PMC4241375

[40] Yang M, Dent M, Lai H, Sun H, Chen Q 2017 Immunization of Zika virus envelope protein domain III induces specific and neutralizing immune responses against Zika virus. Vaccine 35:4287–4294. doi:10.1016/j.vaccine.2017.04.052. 28669618 PMC5546088

[41] Zlatkovic J, Tsouchnikas G, Jarmer J, Koessl C, Stiasny K, Heinz FX 2013 Aluminum hydroxide influences not only the extent but also the fine specificity and functional activity of antibody responses to tick-borne encephalitis virus in mice. J Virol 87:12187–12195. doi:10.1128/JVI.01690-13. 24006434 PMC3807910

[42] Jarmer J, Zlatkovic J, Tsouchnikas G, Vratskikh O, Strauß J, Aberle JH, Chmelik V, Kundi M, Stiasny K, Heinz FX 2014 Variation of the specificity of the human antibody responses after tick-borne encephalitis virus infection and vaccination. J Virol 88:13845–13857. doi:10.1128/JVI.02086-14. 25253341 PMC4248988

[43] Didierlaurent AM, Morel S, Lockman L, Giannini SL, Bisteau M, Carlsen H, Kielland A, Vosters O, Vanderheyde N, Schiavetti F, Larocque D, Van MM, Garcon N 2009 AS04, an aluminum salt- and TLR4 agonist-based adjuvant system, induces a transient localized innate immune response leading to enhanced adaptive immunity. J Immunol 183:6186–6197. doi:10.4049/jimmunol.0901474. 19864596

[44] Garcon N, Di PA 2017 From discovery to licensure, the adjuvant system story. Hum Vaccin Immunother 13:19–33. doi:10.1080/21645515.2016.1225635. 27636098 PMC5287309

[45] Ko EJ, Lee YT, Kim KH, Lee Y, Jung YJ, Kim MC, Lee YN, Kang T, Kang SM 2017 Roles of aluminum hydroxide and monophosphoryl lipid A adjuvants in overcoming CD4+ T cell deficiency to induce isotype-switched IgG antibody responses and protection by T-dependent influenza vaccine. J Immunol 198:279–291. doi:10.4049/jimmunol.1600173. 27881702 PMC5173400

[46] Moon SH, Shin EC, Noh YW, Lim YT 2015 Evaluation of hyaluronic acid-based combination adjuvant containing monophosphoryl lipid A and aluminum salt for hepatitis B vaccine. Vaccine 33:4762–4769. doi:10.1016/j.vaccine.2015.08.006. 26271830

[47] Apostolico JS, Boscardin SB, Yamamoto MM, De Oliveira-Filho JN, Kalil J, Cunha-Neto E, Rosa DS 2016 HIV envelope trimer specific immune response is influenced by different adjuvant formulations and heterologous prime-boost. PLoS One 11:E0145637. doi:10.1371/journal.pone.0145637. 26727218 PMC4699765

[48] Block OK, Rodrigo WW, Quinn M, Jin X, Rose RC, Schlesinger JJ 2010 A tetravalent recombinant dengue domain III protein vaccine stimulates neutralizing and enhancing antibodies in mice. Vaccine 28:8085–8094. doi:10.1016/j.vaccine.2010.10.004. 20959154

[49] Zhao H, Jiang T, Zhou XZ, Deng YQ, Li XF, Chen SP, Zhu SY, Zhou X, Qin ED, Qin CF 2014 Induction of neutralizing antibodies against four serotypes of dengue viruses by MixBiEDIII, a tetravalent dengue vaccine. PLoS One 9:E86573. doi:10.1371/journal.pone.0086573. 24466156 PMC3897746

[50] Yang M, Lai H, Sun H, Chen Q 2017 Virus-like particles that display Zika virus envelope protein domain III induce potent neutralizing immune responses in mice. Sci Rep 7:7679. doi:10.1038/s41598-017-08247-9. 28794424 PMC5550446

[51] Wu Y, Li S, Du L, Wang C, Zou P, Hong B, Yuan M, Ren X, Tai W, Kong Y, Zhou C, Lu L, Zhou X, Jiang S, Ying T 2017 Neutralization of Zika virus by germline-like human monoclonal antibodies targeting cryptic epitopes on envelope domain III. Emerg Microbes Infect 6:E89. doi:10.1038/emi.2017.79. 29018252 PMC5658772

[52] Keeffe JR, Van Rompay KKA, Olsen PC, Wang Q, Gazumyan A, Azzopardi SA, Schaefer-Babajew D, Lee YE, Stuart JB, Singapuri A, Watanabe J, Usachenko J, Ardeshir A, Saeed M, Agudelo M, Eisenreich T, Bournazos S, Oliveira TY, Rice CM, Coffey LL, Macdonald MR, Bjorkman PJ, Nussenzweig MC, Robbiani DF 2018 A combination of two human monoclonal antibodies prevents Zika virus escape mutations in non-human primates. Cell Rep 25:1385–1394. doi:10.1016/j.celrep.2018.10.031. 30403995 PMC6268006

[53] National Research Council. 2011 Guide for the care and use of laboratory animals, 8th ed National Academies Press, Washington, DC.

[54] Tai W, Wang Y, Fett CA, Zhao G, Li F, Perlman S, Jiang S, Zhou Y, Du L 2017 Recombinant receptor-binding domains of multiple Middle East respiratory syndrome coronaviruses (MERS-CoVs) induce cross-neutralizing antibodies against divergent human and camel MERS-CoVs and antibody escape mutants. J Virol 91:e01651-16. doi:10.1128/JVI.01651-16. 27795425 PMC5165220

[55] Zhang N, Channappanavar R, Ma C, Wang L, Tang J, Garron T, Tao X, Tasneem S, Lu L, Tseng CT, Zhou Y, Perlman S, Jiang S, Du L 2016 Identification of an ideal adjuvant for receptor-binding domain-based subunit vaccines against Middle East respiratory syndrome coronavirus. Cell Mol Immunol 13:180–190. doi:10.1038/cmi.2015.03. 25640653 PMC4786625

[56] Ma C, Wang L, Tao X, Zhang N, Yang Y, Tseng CT, Li F, Zhou Y, Jiang S, Du L 2014 Searching for an ideal vaccine candidate among different MERS coronavirus receptor-binding fragments—the importance of immunofocusing in subunit vaccine design. Vaccine 32:6170–6176. doi:10.1016/j.vaccine.2014.08.086. 25240756 PMC4194190

[57] Du L, Zhao G, Yang Y, Qiu H, Wang L, Kou Z, Tao X, Yu H, Sun S, Tseng CT, Jiang S, Li F, Zhou Y 2014 A conformation-dependent neutralizing monoclonal antibody specifically targeting receptor-binding domain in Middle East respiratory syndrome coronavirus spike protein. J Virol 88:7045–7053. doi:10.1128/JVI.00433-14. 24719424 PMC4054355

[58] Koide F, Goebel S, Snyder B, Walters KB, Gast A, Hagelin K, Kalkeri R, Rayner J 2016 Development of a Zika virus infection model in cynomolgus macaques. Front Microbiol 7:2028. doi:10.3389/fmicb.2016.02028. 28066354 PMC5165249

[59] Xia S, Xu W, Wang Q, Wang C, Hua C, Li W, Lu L, Jiang S 2018 Peptide-based membrane fusion inhibitors targeting HCoV-229E spike protein HR1 and HR2 domains. IJMS 19:487. doi:10.3390/ijms19020487. PMC5855709 29415501

[60] Chou TC 2006 Theoretical basis, experimental design, and computerized simulation of synergism and antagonism in drug combination studies. Pharmacol Rev 58:621–681. doi:10.1124/pr.58.3.10. 16968952

[61] Hachiya A, Reeve AB, Marchand B, Michailidis E, Ong YT, Kirby KA, Leslie MD, Oka S, Kodama EN, Rohan LC, Mitsuya H, Parniak MA, Sarafianos SG 2013 Evaluation of combinations of 4′-ethynyl-2-fluoro-2′-deoxyadenosine with clinically used antiretroviral drugs. Antimicrob Agents Chemother 57:4554–4558. doi:10.1128/AAC.00283-13. 23796932 PMC3754316

[62] Lin B, He S, Yim HJ, Liang TJ, Hu Z 2016 Evaluation of antiviral drug synergy in an infectious HCV system. Antivir Ther 21:595–603. doi:10.3851/IMP3044. 27035622 PMC5580926

[63] Yu Y, Deng YQ, Zou P, Wang Q, Dai Y, Yu F, Du L, Zhang NN, Tian M, Hao JN, Meng Y, Woo JFW, Yuen KY, Qin CF, Jiang S, Lu L 2017 A peptide-based viral inactivator inhibits Zika virus infection in pregnant mice and fetuses. Nat Commun 8:15672. doi:10.1038/ncomms15672. 28742068 PMC5537589

[64] Tai W, Voronin D, Chen J, Bao W, Kessler DA, Shaz B, Jiang S, Yazdanbakhsh K, Du L 2019 Transfusion-transmitted Zika virus infection in pregnant mice leads to broad tissue tropism with severe placental damage and fetal demise. Front Microbiol 10:29. doi:10.3389/fmicb.2019.00029. 30728813 PMC6351479

